# Associations Between Miscarriage and Postpartum Depression, Perceived Social Support, and Medical Needs in Pregnant Women: A Single-Center Retrospective Study

**DOI:** 10.62641/aep.v54i3.2201

**Published:** 2026-06-15

**Authors:** Nana Kong, Xiaoyuan Wu, Cui Li, Ruifang Cao, Zhuo Chen, Xiaobing Guo, Jing Zhang

**Affiliations:** ^1^Obstetrics Department, Xingtai People’s Hospital, 054001 Xingtai, Hebei, China; ^2^Operating Room, Xingtai People’s Hospital, 054001 Xingtai, Hebei, China

**Keywords:** fetal loss, the mother, depressive symptoms, social support, psychological factors

## Abstract

**Background:**

Fetal loss constitutes a major obstetric adverse outcome, and is frequently followed by marked psychological distress; the prevalence of depressive symptoms after fetal loss is substantially higher, and this elevation is intertwined with psychosocial determinants whose clinical profiles and intervention targets await systematic synthesis. This study examines the clinical characteristics and psychosocial determinants of maternal depression following fetal loss, aiming to inform targeted psychological support strategies.

**Methods:**

This retrospective study included 200 mothers following fetal loss (fetal loss group) and 200 mothers after normal delivery (term delivery group), selected via 1:1 nearest-neighbour propensity score matching (PSM) between June 2022 and October 2025. At 42 days post-event, participants in both groups completed the Edinburgh Postnatal Depression Scale (EPDS), Hospital Anxiety and Depression Scale (HADS), Multidimensional Scale of Perceived Social Support (MSPSS), and the Olson Marital Quality Questionnaire (ENRICH). Depression was defined as EPDS ≥13. Depression prevalence and scale scores were compared between groups, and multivariable logistic regression identified risk factors associated with depression. Changes in EPDS score reduction (Δ = score at 3 months postpartum – baseline score) was compared between those who received and those who did not receive clinical management.

**Results:**

Following PSM, baseline characteristics were well-balanced between the two groups (*p* > 0.05). The prevalence of depression was significantly higher among women with the fetal loss than among those with term delivery group (35.0% vs. 8.50%, *p* < 0.05). Multivariate analysis identified fetal loss as an independent predictor of depression (odds ratio (OR) = 2.84, 95% confidence interval (CI): 1.96–4.12). EPDS scores were significantly higher in the fetal loss group than in the term delivery group (13.1 ± 4.0 vs. 8.5 ± 2.0, *p* < 0.001). The predominant symptoms included persistent low mood (87.1%), insomnia (75.7%), guilt or self-blame (68.6%) and fear or avoidance of future pregnancy (62.9%). Within the fetal loss group, Low social support (OR = 3.15), marital dissatisfaction (OR = 2.43), ≥2 abortions (OR = 1.98), and lack of clinical management (OR = 2.27) were independently predicted depression. Only 27.6% of affected mothers received treatment, and this was associated with significantly greater improvement in EPDS scores (Δ = –5.2 ± 2.4 vs. –1.9 ± 2.0, *p* < 0.001).

**Conclusions:**

Fetal loss is associated with a substantially increased risk of maternal depression, characterized by self-blame and fear of future pregnancy. Modifiable factors including low social support and absent professional care, are associated with more persistent depressive symptoms. These Findings support the intervention integration for high-risk mothers, although further validation is required.

## Introduction

Fetal loss, defined as spontaneous pregnancy loss at or after 12 weeks of gestation (including inevitable abortion, missed abortion, and stillbirth), represents one of the most profound adverse life events experienced by women of reproductive age [1]. It not only terminates a pregnancy but also imposes substantial psychological trauma on affected women. During the post-loss period, women must not only cope with physical recovery, but also with complex grief reactions, placing them at a markedly increased risk of mental health problems [2, 3, 4].

A growing body of clinical observations indicates that mothers who experience fetal loss are at a high risk for depressive disorder. They exhibit a significantly higher prevalence of core depressive symptoms—including persistent low mood and anhedonia compared with uncomplicated term delivery [5, 6]. If such psychological distress is not recognized and appropriately managed, it may fail to resolve, which thereby impairing the mother’s well-being, social engagement, and marital dynamics, and also poses long-term implications for subsequent reproductive decisions and mental health in future pregnancies [7, 8].

Although the association between fetal loss and increased depression risk is well established, substantial gaps remain in both clinical understanding and practice [9]. First, specific depressive features—particularly pronounced feelings of self-blame/guilt and fear of a future pregnancy—have been described in anecdotal reports and qualitative studies, but have not been systematically quantified in controlled investigations. Consequently, their prevalence, distinguishing characteristics, and clinical relevance in post-loss depression remain unclear. Second, depression in this population is closely associated with multiple psychosocial factors, including social support, marital quality, and prior obstetric history. However, how these factors interact and which of them represent the most potent and modifiable intervention targets remain to be clarified, especially after rigorous control of confounders. Third, there is a paucity of real-world evidence regarding the actual uptake of mental health services among this vulnerable population and the effectiveness of those interventions when delivered in routine care.

This retrospective cohort study, employing propensity score matching (PSM), systematically explored the clinical characteristics, related psychosocial factors, and clinical management status of maternal depression after fetal loss, and analysed the association between receiving clinical management and symptom improvement. We hypothesized that: compared with mothers following uncomplicated term delivery, those experiencing fetal loss would exhibit a distinct clinical phenotype characterized by a higher proportion of self-blame/guilt and fear of subsequent pregnancy. Fetal loss would be an independent correlate of subsequent depression. In within the fetal loss group, depression symptoms were expected to be significantly associated with low social support, poor marital satisfaction, a history of recurrent abortion and the absence of professional medical care. Among mothers with fetal loss who screened positive for depressive symptoms, those who received systematic clinical management would show significantly greater symptom improvement than those who did not. This study aims to provide robust evidence to support early identification of depression risk after fetal loss in clinical practice, to inform individualized psychological support strategies, and ultimately improve mental health outcomes in this vulnerable population.

## Materials and Methods

### Study Design

**Fig. 1.**
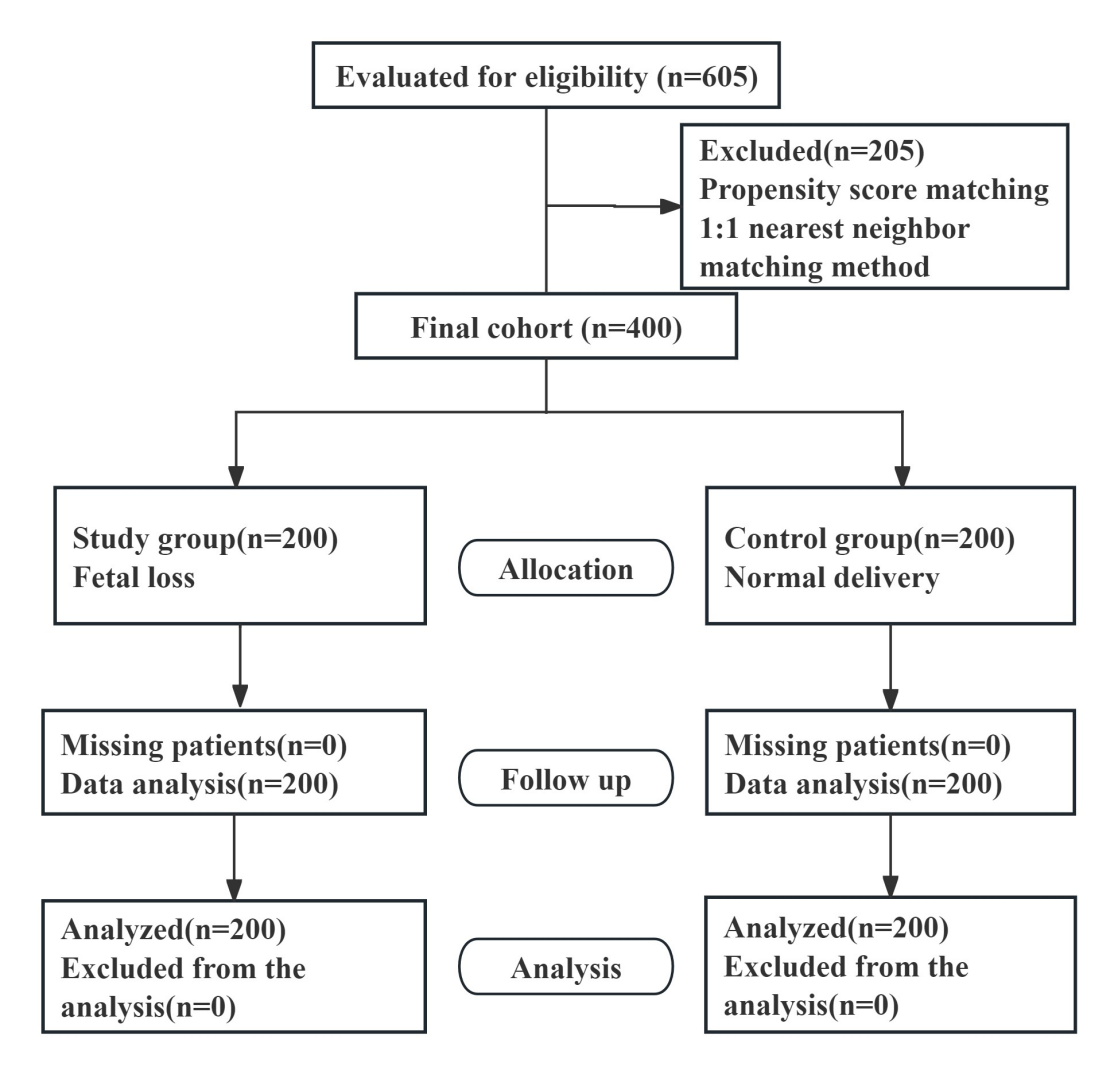
**Flow chart of the study**.

A retrospective cohort design was adopted. Clinical records were retrieved from our hospital’s obstetric database for mothers with a diagnosis of fetal loss between June 2022 and October 2025, and for matched term deliverys with normal deliveries during the same period. To minimise potential confounding bias between the two groups, a 1:1 PSM was used to select 200 mothers with fetal loss were included in the fetal loss group and 200 mothers with normal delivery were included in the term delivery group. To compare the differences in postpartum depression symptoms, psychosocial factors and received clinical management needs between fetal loss mothers and normal delivery mothers. The design operation process is presented in Fig. 1.

### Inclusion Criteria

Fetal loss group [10, 11]: (1) Fetal loss (defined as spontaneous pregnancy loss at ≥12 weeks of gestation, including inevitable abortion, missed abortion, and stillbirth) was diagnosed at 12 weeks of gestation or above; (2) age ≥18 years old; (3) receiving standard postpartum follow-up and psychological assessment 42 days after fetal loss; (4) Complete medical records; (5) no history of mental illness.

Term delivery group: (1) Age ≥18 years old, full-term (gestational age ≥37 weeks) normal delivery (vaginal delivery or caesarean section) in our hospital during the same period, with good maternal and infant outcomes; (2) the relevant scales were evaluated 42 days after delivery, and the clinical data were complete. (3) no history of fetal loss or mental illness.

### Exclusion Criteria

(1) Those who elected to terminate pregnancy due to severe fetal malformation (to reduce the complex psychological confusion caused by the decision itself); (2) patients with a history of severe psychiatric disorders (such as schizophrenia, bipolar disorder) or a prior diagnosis of depression before the event; (3) Complicated with serious physical diseases (such as malignant tumors, serious heart, liver and kidney diseases); (4) communication barriers, unable to complete the scale assessment; (5) taking antidepressants, antipsychotic drugs and other drugs during pregnancy or postpartum that may affect mood assessment [4].

### Sample Size Calculation

Based on pilot data from the present study investigation, supported by ranges reported in previous literature [12, 13], the expected prevalence of depression rate was approximately 30% in the fetal loss group and 10% in the term delivery group. The test level α = 0.05 (two-sided), the test power 1-β = 0.80, and the sample size was estimated by PASS 15.0 statistical software (NCSS, LLC, Kaysville, UT, USA). Employing the formula for comparing two independent proportions, the initial calculation mandated a minimum of 170 per group. Considering the possible missing data in the retrospective design and the sample attrition after PSM matching, the sample size was appropriately increased by 20%. Finally, 200 cases in per group were included in the final analysis after PSM matching, thereby meeting the requisite statistical power.

### Clinical Management Received

This retrospective study examined the spectrum of clinician-delivered management provided to peripartum women who screened positive for depressive symptoms (Edinburgh Postnatal Depression Scale (EPDS) ≥13) during routine follow-up following fetal loss or term delivery. Documented in medical records, these clinical interventions were driven by current clinical guidelines [14, 15] and tailored to individual patient conditions, and included: Psychological counseling (e.g., supportive psychotherapy, grief counseling); Psychoeducation (e.g., information on depressive symptoms, coping strategies, and recovery); Referral to psychiatric services for evaluation and pharmacotherapy, when indicated. These received clinical management strategies are supported by empirical evidence. Psychological clinical management—including interpersonal psychotherapy and mindfulness-based stress reduction—have been shown to reduce depressive symptoms following perinatal loss [16, 17], and pharmacotherapy is an established treatment for moderate to severe postnatal depression [14]. Despite the existence of effective options, substantial disparities in clinical uptake persist [18]. All approaches were delivered and documented as integral components of routine care. Based on these retrospective data, the present study analysed the association between receipt of such approaches and the magnitude of symptom improvement.

### Evaluation Indicators

By consulting the hospital electronic medical record system and special psychological assessment files, the data of the following evaluation indicators were retrospectively extracted and collected.

#### Baseline Data

Demographic characteristics (age, education level, family monthly income, marital status, occupation and residence) and pregnancy-related variables (gestational age, parity, previous history of abortion, pregnancy complications, assisted reproduction) were collected.

#### Main Outcome Measures

Depressive symptoms: EPDS was used for evaluation. All participants completed the EPDS at 42 days post-loss or postpartum (baseline). EPDS is a 10-item scale evaluates mood over the past 7 days, with each item scored from 0 to 3, yielding a total score ranging from 0 to 30. A cut-off score of ≥13 was used as the cut-off for probable depression, a threshold with established high sensitivity that is widely applied in perinatal and bereaved populations [19, 20]. Although this cut-off has not been less rigorously validated specifically in post-loss populations, it remains the most frequently used screening threshold in perinatal bereavement research. The EPDS was designated as the primary outcome measure. Depression was defined as an EPDS score ≥13 at 42 days, and this binary variable served as the dependent variable in all logistic regression analyses. For participants who screened positive (EPDS ≥13), the EPDS was reassessed at 3 months post-loss or postpartum to evaluate changes in symptom severity. In addition to the EPDS, the Hospital Anxiety and Depression Scale (HADS) was administered. The HADS-D (depression subscale) was used as a secondary, continuous measure of depressive symptom severity to complement EPDS findings and to facilitate comparisons with non-perinatal populations. while HADS-A (anxiety subscale) provided complementary information on anxiety symptoms, offering a broader characterization of psychological distress. In the present sample, the EPDS demonstrated good internal consistency, with a Cronbach’s α coefficient of 0.87.

#### Secondary Outcome Measures

(1) Anxiety and depression: HADS [21] was administered at the 42-day. It consists of 14 items, evenly divided into anxiety (HADS-A) and depression (HADS-D) subscales. Each item is rated on a 0–3 scale, with each subscale score ranging from 0 to 21. Higher scores indicate greater symptoms severity. In this study, the Cronbach’s α coefficients were 0.83 for HADS-A and 0.81 for HADS-D, indicating good internal consistency.

(2) Social support: Multidimensional Scale of Perceived Social Support (MSPSS) [22] was administered at the 42-day time point. It includes 12 items evaluating support from family, friends, and significant others. Items are rated on a 7-point Likert scale, yielding a total score ranging from 12 to 84. Higher scores indicate greater perceived social support. To facilitate clinical interpretation and identify high-risk subgroups, MSPSS scores were dichotomized; “low social support” was defined as a total score within the lowest quartile of the study population (<25th percentile, i.e., ≤34 in this cohort). The same cut-off was applied to both groups to ensure comparability. The Cronbach’s α for the MSPSS in the current sample was 0.91, indicating excellent internal consistency.

(3) The marital satisfaction subscale of Olson Marital Quality Questionnaire (ENRICH) [23] was used for evaluation at the 42-day time point. This 10-item subscale evaluates the level of satisfaction with the marital relationship. Items are rated on a 5-point Likert scale, with total scores ranging from 10 to 50. Higher scores indicate greater marital satisfaction. In the absence of a validated clinical threshold for this population, “poor marital satisfaction” was defined as a score within the lowest quartile of the study population (≤29 in this cohort). This cut-off was selected to identify the most vulnerable individuals while ensuring comparability between groups. The Cronbach’s α for the ENRICH marital satisfaction subscale was 0.85, indicating good internal consistency.

(4) Received clinical management situation and effect evaluation: Based on medical records, the proportion of depression-positive mothers (including both the study and term delivery groups) who received a clinical management between 42 days post-loss or postpartum and 3 months postpartum was calculated. Clinical management effectiveness was evaluated using the EPDS score change (△), where △ = follow-up score at 3 months postpartum – baseline score (at 42 days post-loss or postpartum). A more negative △ value indicates greater improvement in depressive symptoms.

### Statistical Analysis

Retrospective data were analysed using SPSS 26.0 (IBM Corp., Armonk, NY, USA). Continuous variables with a normal distribution are presented as mean ± standard deviation and compared using the independent samples *t*-test. Non-normally distributed data were presented as median (interquartile range, IQR) and analysed with the Mann-Whitney U test. Categorical variables are presented as counts (percentages) [n (%)] and were compared using the chi-square test or Fisher’s exact test [24]. To minimise the baseline confounding factors, age, gravidity, parity, previous abortion history, education level, family monthly income, residence, assisted reproduction, marital status and pregnancy-related complications were used as matching variables [25, 26, 27]. The fetal loss and term delivery groups were matched 1:1 using nearest neighbor matching with a caliper of 0.02 was applied. Balance was achieved when the standardized mean difference (SMD) was <0.1. Variables with *p* < 0.1 in univariate analysis along with “fetal loss status” were entered into a multivariate logistic regression model to calculate odds ratios (ORs) and 95% confidence intervals (CIs). The dependent variable was depression status, defined as EPDS ≥13 at 42 days. Within the fetal loss group, a second multivariate logistic regression was conducted to identify independent risk factors for depression using the same EPDS-defined depression status as the outcome. Among depressed mothers in the fetal loss group, the reduction in EPDS scores (△) were compared between those who received clinical management and those who did not using an independent samples *t*-test. To assess potential confounding, baseline demographic and clinical characteristics were compared between these subgroups among all depression-positive mothers (n = 87), using independent samples *t*-tests or χ^2^ tests as appropriate. In addition, analysis of covariance (ANCOVA) was performed with baseline EPDS score as a covariate to estimate the adjusted between-group difference in ΔEPDS. Given the non-randomized, retrospective design, no causal inference can be drawn from this comparison. To evaluate the robustness of the findings, MSPSS and ENRICH were also entered as continuous variables in the same multivariate logistic regression models. All statistical tests were two-sided, with *p* < 0.05 considered statistically significant.

## Results

### Comparison of Baseline Data

Initially, 605 patients were included in the study, split into 255 (fetal loss group) and 350 (term delivery group). Prior to PSM, the two groups differed significantly in multiple key demographic and obstetric characteristics. The fetal loss group had older mothers and a higher number of pregnancies, had a higher proportion of multiparas women, a significantly higher proportion of previous abortions (especially ≥2), had a lower monthly family income, and had significant differences in the proportion of assisted reproduction, marital status, pregnancy-related complications, and occupational status (all *p* values < 0.05). These systematic differences suggest that, if unadjusted, these factors are likely to act as confounders that interfere with the judgment of the relationship between the core exposure of “fetal loss” and depressive outcomes. After 1:1 PSM, 200 mothers in the fetal loss group were successfully matched to 200 mothers in the term delivery group with similar baseline characteristics. Among the 200 mothers in the fetal loss group after PSM, the distribution of loss types was as follows: missed abortion (n = 98, 49.0%), inevitable abortion (n = 62, 31.0%), and stillbirth (n = 40, 20.0%). Both groups had *p* values greater than 0.05 for all baseline measures compared. As shown in Table 1. These results demonstrate that PSM effectively balanced the measured confounding factors between the treatment and term delivery groups, thereby enhancing group comparability.

**Table 1.** **Patient baseline characteristics before and after PSM**.

Indicators		Before PSM				After PSM			
		Fetal Loss Group	Term Delivery Group	Statistic	*p* value	Fetal Loss Group	Term Delivery Group	Statistic	*p* value
		(n = 255)	(n = 350)			(n = 200)	(n = 200)		
Age (years), mean ± SD		30.2 ± 4.8	28.9 ± 4.1	*t* = 3.48	0.001	30.1 ± 4.7	30.3 ± 4.3	*t* = –0.42	0.674
Age group, n (%)				χ^2^ = 5.43	0.020			χ^2^ = 0.14	0.704
	<35 years	207 (81.2)	308 (88.0)			163 (81.5)	160 (80.0)		
	≥35 years	48 (18.8)	42 (12.0)			37 (18.5)	40 (20.0)		
Gravidity, Median (IQR)		1.5 (1.0, 2.9)	1.8 (1.0, 2.9)	*Z*= −1.18	0.382	1.5 (1.0, 2.9)	1.8 (1.0, 2.9)	*Z* = −0.13	0.854
Parity, n (%)				χ^2^ = 13.28	<0.001			χ^2^ = 0.01	0.917
	Nulliparous	163 (63.9)	271 (77.4)			127 (63.5)	128 (64.0)		
	Multiparous	92 (36.1)	79 (22.6)			73 (36.5)	72 (36.0)		
Prior abortions, n (%)				χ^2^ = 16.47	<0.001			χ^2^ = 0.10	0.949
	0	125 (49.0)	227 (64.8)			100 (50.0)	98 (49.0)		
	1	79 (31.0)	83 (23.7)			62 (31.0)	65 (32.5)		
	≥2	51 (20.0)	40 (11.4)			38 (19.0)	37 (18.5)		
Pregnancy-related comorbidities, n (%)									
	Hypertension	25 (9.8%)	18 (5.1%)	χ^2^ = 4.86	0.027	18 (9.0%)	16 (8.0%)	χ^2^ = 0.12	0.729
	Diabetes	18 (7.1%)	13 (3.7%)	χ^2^ = 3.39	0.066	13 (6.5%)	12 (6.0%)	χ^2^ = 0.05	0.823
	Thyroid disease	30 (11.8%)	20 (5.7%)	χ^2^ = 7.13	0.008	20 (10.0%)	19 (9.5%)	χ^2^ = 0.03	0.865
Marital status, n (%)				χ^2^ = 7.32	0.007			χ^2^ = 0.08	0.778
	Married/Stable cohabitation	204 (80.0%)	308 (88.0%)			170 (85.0%)	172 (86.0%)		
	Unmarried/Divorced/Widowed	51 (20.0%)	42 (12.0%)			30 (15.0%)	28 (14.0%)		
Education level, n (%)				χ^2^ = 1.86	0.173			χ^2^ = 0.05	0.831
	College degree or above	172 (67.5)	254 (72.6)			136 (68.0)	134 (67.0)		
	Below college degree	83 (32.5)	96 (27.4)			64 (32.0)	66 (33.0)		
Occupation, n (%)				χ^2^ = 7.17	0.007			χ^2^ = 0.05	0.823
	Employed	180 (70.6%)	280 (80.0%)			150 (75.0%)	152 (76.0%)		
	Unemployed	75 (29.4%)	70 (20.0%)			50 (25.0%)	48 (24.0%)		
Monthly household income, n (%)				χ^2^ = 7.90	0.005			χ^2^ = 0.18	0.668
	< ¥10,000 (< 1389 USD)	87 (34.1)	83 (23.7)			66 (33.0)	62 (31.0)		
	≥ ¥10,000 (≥ 1389 USD)	168 (65.9)	267 (76.3)			134 (67.0)	138 (69.0)		
Residence, n(%)				χ^2^ = 3.25	0.072			χ^2^ = 0.11	0.742
	Urban	181 (71.0)	271 (77.4)			143 (71.5)	140 (70.0)		
	Rural	74 (29.0)	79 (22.6)			57 (28.5)	60 (30.0)		
Assisted reproductive technology use, n (%)		28 (11.0)	14 (5.4)	χ^2^ = 11.13	0.001	20 (10.0)	18 (9.0)	χ^2^ = 0.11	0.742

Note: PSM, propensity score matching; IQR, interquartile range; Exchange rate: 1 USD ≈ 7.20 CNY (based on the average exchange rate during the study period).

### Incidence and Risk Analysis of Depression

#### Comparison of Depression Incidence

Table 2 demonstrates that the depression incidence in the fetal loss group (35.0%) was significantly higher than in the term delivery group (8.5%), with a highly significant difference (*p* < 0.001). The results showed that fetal loss was significantly associated with higher odds of maternal depression.

**Table 2.** **Comparison of depression incidence between the two groups [n (%)]**.

Group	n	Positive cases (n)	Incidence (%)	Statistic	*p* value
Fetal Loss Group	200	70	35.0	χ^2^ = 42.631	<0.001
Term Delivery Group	200	17	8.5		

Note: χ^2^ test was applied.

### Comparison of Total EPDS Scores

As shown in Table 3, after adjustment for baseline confounding factors, the total EPDS score was significantly higher in the fetal loss group (13.1 ± 4.0) than in term delivery group (8.5 ± 2.0), with a highly significant difference (*p* < 0.001). The mean score of the fetal loss group (13.1) exceeded the commonly used EPDS depression screening cutoff of 13. These findings suggest that the overall severity of depressive symptoms in mothers with fetal loss had reached a level warranting clinical attention and clinical management by 42 days postpartum. The effect size (Cohen’s d = 1.08) suggests that the impact of fetal loss on maternal depressive symptom severity was not only statistically significant but also clinically substantial.

**Table 3.** **Comparison of EPDS total scores between groups ($\bar{x}$ ± s)**.

Group	n	EPDS	Effect size (Cohen’s d)	Statistic	*p* value
Fetal Loss Group	200	13.1 ± 4.0	1.08	*t* = 12.84	<0.001
Term Delivery Group	200	8.5 ± 2.0	-	-	-

Note: Independent samples *t*-test was applied. Effect size interpretation: —Cohen’s d— ≥0.2 (small), ≥0.5 (medium), ≥0.8 (large); EPDS, Edinburgh Postnatal Depression Scale.

#### Univariate and Multivariate Logistic Regression Analysis of Influencing Factors of Depression

Univariate logistic regression analysis identified 7 variables associated with the occurrence of maternal depressive symptom. The odds of maternal depression were 4.15 times higher in the fetal loss group than in the normal delivery group (95% CI: 2.43–7.08), representing the strongest association observed. Low level of social support, poor marital satisfaction, history of abortion ≥2 times, family monthly income <10,000 CNY (approximately 1389 USD), age ≥35 years old and education attainment below junior college level. Variables with *p* < 0.1 in the univariate analysis were incorporated into a multivariate logistic regression model following adjustment for confounding effects. The findings are presented in Table 4. Fetal loss remained independently associated with maternal depression after adjustment (adjusted odds ratio, aOR = 2.84, 95% CI: 1.96–4.12, *p* < 0.001). After adjustment, the magnitude of the association was slightly attenuated, it remained highly significant. Low social support (aOR = 3.16, *p* < 0.001), poor marital satisfaction (aOR = 2.44, *p* = 0.001) and history of abortion ≥2 abortions (aOR = 1.98, *p* = 0.016) were identified as independent predictors of depression. However, monthly household income <10,000 CNY (approximately 1389 USD) (*p* = 0.142), age ≥35 years old and education level below college were not significantly associated with depression after multivariable adjusting. This suggests that these factors may indirectly affect depression through interaction with other variables rather than directly independent risk factors. Place of residence was not included in the multivariate model due to a lack of significance in the univariate analysis (*p* > 0.10). In a sensitivity analysis treating MSPSS and ENRICH scores as continuous variables, each 1-point increase remained significantly associated with lower odds of depression (MSPSS: adjusted OR = 0.94, 95% CI: 0.91–0.97, *p* < 0.001; ENRICH: adjusted OR = 0.95, 95% CI: 0.92–0.98, *p* = 0.001), confirming the robustness of the findings.

**Table 4.** **Univariate and multivariate logistic regression analyses for depressive symptoms**.

Variable	Univariate analysis		Multivariate analysis	
	OR (95% CI)	*p* value	aOR (95% CI)	*p* value
Fetal Loss Group (Fetal Loss)	4.15 (2.43–7.08)	<0.001	2.84 (1.96–4.12)	<0.001
Low Social Support	3.39 (2.01–5.73)	<0.001	3.16 (1.87–5.35)	<0.001
Poor Marital Satisfaction	2.59 (1.53–4.38)	<0.001	2.44 (1.44–4.13)	0.001
Prior Abortions ≥2	2.34 (1.34–4.07)	0.003	1.98 (1.13–3.46)	0.016
Monthly Income < ¥10,000 (< 1389 USD)	2.05 (1.17–3.61)	0.011	1.51 (0.88–2.58)	0.142
Age ≥35 Years	1.67 (0.92–3.01)	0.073	1.32 (0.74–2.36)	0.352
Education (Below College)	1.62 (0.94–2.81)	0.089	1.28 (0.74–2.21)	0.381
Residence (Rural)	1.55 (0.89–2.72)	0.12	-	-

Note: OR, odds ratio; aOR, adjusted odds ratio; CI, confidence interval. Variables with *p* < 0.1 in univariate analysis were incorporated into the multivariate logistic regression model; “-” signifies variables excluded from the final model; Exchange rate: 1 USD ≈ 7.20 CNY (based on the average exchange rate during the study period).

### Score of Psychological and Social Function Scale

As shown in Table 5, the score results of the Psychological and Social Functioning Scale (PSFC) indicated that the fetal loss group experienced significant and serious disadvantages in both dimensions of emotional state and psychosocial resources. HADS-A and HADS-D scores were significantly higher in the fetal loss group than in the term delivery group (both *p* < 0.001), with an extremely large effect size (Cohen’s d >1.0). MSPSS and ENRICH scores were significantly lower in the fetal loss group compared to the term delivery group. In particular, the difference effect size of social support was extremely large (d = –2.44) was very large, which strongly suggested that the external resources actually felt or available to this group were seriously insufficient when they needed support. The results indicate that mothers with fetal loss are subject to extensive psychosocial impairment, characterised by coexisting severe anxiety and depression alongside markedly diminished social support system.

**Table 5.** **Comparison of psychological and social functioning scale scores between the two groups ($\bar{x}$ ± s)**.

Scale	Fetal Loss Group (n = 200)	Term Delivery Group (n = 200)	Effect size (Cohen’s d)	Statistic	*p* value
HADS-A	10.2 ± 3.8	6.6 ± 3.1	1.05	10.49	<0.001
HADS-D	11.8 ± 4.1	7.3 ± 3.5	1.20	11.79	<0.001
MSPSS	42.5 ± 10.6	65.8 ± 8.4	–2.44	–24.24	<0.001
ENRICH	34.2 ± 9.1	41.5 ± 8.3	–0.83	–8.51	<0.001

Note: HADS-A/D, Hospital Anxiety and Depression Scale-Anxiety/Depression subscale; MSPSS, Perceived Social Support Scale; ENRICH, Marital Satisfaction Scale. Higher HADS-A/D scores indicate more severe anxiety/depressive symptoms. Higher MSPSS and ENRICH scores indicate greater perceived social support and higher marital satisfaction, respectively. Comparisons were performed using independent samples *t*-tests. The absolute value of Cohen’s d was interpreted as: 0.2 (small), 0.5 (medium), and 0.8 (large).

### Clinical Manifestations of Depressive Symptoms

Among depression-positive mothers, a comparison of clinical symptoms between the two groups (fetal loss group, n = 70; term delivery group, n = 17) is presented in Table 6. The results showed that there were no statistically significant differences in the reported rates of typical depressive symptoms (e.g., persistent low mood, nocturnal insomnia, loss of interest, difficulty concentrating; all *p* > 0.05). However, there were significant group-specific differences in core cognitive and trauma-related symptoms. Mothers in the fetal loss group (following pregnancy loss) reported significantly higher rates of self-blame or guilt (68.6% vs. 35.3%, *p *= 0.009) and fear or avoidance of future pregnancy (62.9% vs. 17.6%, *p *= 0.001) compared to depressed mothers in the term delivery group (after term delivery).

**Table 6.** **Comparison of primary clinical symptoms in depression-positive mothers between the fetal loss group (n = 70) and the term delivery group (n = 17) [n (%)]**.

Clinical Symptom	Fetal Loss Group (n = 70)	Term Delivery Group (n = 17)	Statistic	*p* value
Persistent Low Mood	61 (87.1%)	15 (88.2%)	0.02	0.89
Insomnia at Night	53 (75.7%)	13 (76.5%)	0.01	0.95
Feelings of Guilt or Self-Blame	48 (68.6%)	6 (35.3%)	FET	0.009
Fear or Avoidance of Future Pregnancy	44 (62.9%)	3 (17.6%)	FET	0.001
Loss of Interest	40 (57.1%)	9 (52.9%)	0.09	0.76
Difficulty Concentrating	35 (50.0%)	8 (47.1%)	0.05	0.83

Note: FET, Fisher’s exact test. For cells with expected counts <5, FET was used for analysis. Only mothers who screened positive for depression (EPDS ≥ 13) were included in this analysis.

### Association Between Treatment Received and Symptom Improvement

Among all 87 depression-positive mothers (70 in the fetal loss group and 17 in the term delivery group), 24 (27.6%) received systematic clinical management (including psychological support and health education) during follow-up, while 63 (72.4%) did not receive any targeted intervention, indicating a substantial gap in care provision. Baseline characteristics of the received clinical management and no clinical management subgroups were comparable. No significant differences were observed in age, education, monthly income, parity, prior abortion history, or baseline EPDS score (15.0 ± 3.9 vs. 14.5 ± 4.5, *p* = 0.62). Those who received clinical management showed more pronounced improvement in depressive symptoms. As presented in Table 7, the mean EPDS score at the 3-month follow-up was5.2 ± 2.4 points in the clinical management group, which was significantly greater than that of the no clinical management group (1.9 ± 2.0 points), with the difference being statistically significant (*p* < 0.001). After adjustment for baseline EPDS score using ANCOVA, receipt of clinical management remained significantly associated with greater symptom improvement (adjusted mean difference in △EPDS = –3.1, 95% CI: –4.4 to –1.8, *p* < 0.001), consistent with the unadjusted comparison.

**Table 7.** **Comparison of symptom improvement in all depression-positive mothers who did and did not receive clinical management ($\bar{x}$ ± s)**.

Group	n	Baseline EPDS	3-Month EPDS	△EPDS	Statistic	*p* value
Received Clinical Management	24	15.0 ± 3.9	9.5 ± 3.6	–5.2 ± 2.4	5.92	<0.001
No Clinical Management	63	14.5 ± 4.5	12.1 ± 4.3	–1.9 ± 2.0	-	-

Note: △EPDS = 3-Month score - Baseline score. A negative △ value indicates a reduction in depressive symptoms, with a larger absolute value representing greater improvement. An independent samples *t*-test was used to compare △EPDS between groups.

### Factors Associated With Depression in the Fetal Loss Group (Mothers With Fetal Loss)

Univariate analysis indicated that low social support, poor marital satisfaction, previous abortion ≥2 times, no medical management were significantly associated with depression after fetal loss (*p* < 0.1). Monthly household income < ¥10,000 CNY (approximately 1389 USD) was not significantly associated with depression in univariate analysis (*p* = 0.18) and was therefore not entered into the multivariate model. Multivariate logistic regression identified four factors independently associated with depression (Table 8). The strongest association was observed for low social support (aOR = 3.15, 95% CI: 1.85–5.36, *p* < 0.001). Poor marital satisfaction was also significantly increased the probability of depression (aOR = 2.43, 95% CI: 1.41–4.19, *p *= 0.001). Mothers with ≥2 prior abortions had nearly twice the odds of depression (aOR = 1.98, 95% CI: 1.10–3.56, *p* = 0.023), while absence of medical management was associated with 2.3-fold higher odds of persistent depression 2.3-fold (aOR = 2.27, 95% CI: 1.28–4.04, *p *= 0.005). Education level below college was included in the multivariate model based on its univariate *p* value (<0.1), but it did not reach statistical significance after adjustment (aOR = 1.48, 95% CI: 0.86–2.55, *p* = 0.157). Age ≥35 years, assisted reproductive technology use, and monthly household income were not significantly associated with depression in univariate analysis (*p* > 0.1) and were therefore not entered into the final model. In the fetal loss group, continuous MSPSS and ENRICH scores also showed significant protective effects (MSPSS: aOR = 0.93, 95% CI: 0.90–0.96, *p* < 0.001; ENRICH: aOR = 0.94, 95% CI: 0.91–0.97, *p* < 0.001), consistent with the dichotomized results. In conclusion, the prevention and treatment of depression after fetal loss should focus on the three high-risk groups of low social support, marital conflict, and recurrent abortion history, and the risk pathways should be blocked through proactive and systematic medical management.

**Table 8.** **Factors associated with depression in the fetal loss group: univariate and multivariate analyses**.

Variable	Univariate analysis		Multivariate analysis	
	OR (95% CI)	*p* value	aOR (95% CI)	*p* value
Low Social Support	3.40 (2.01–5.75)	<0.001	3.15 (1.85–5.36)	<0.001
Poor Marital Satisfaction	2.60 (1.54–4.39)	<0.001	2.43 (1.41–4.19)	0.001
Prior Abortions ≥2	2.32 (1.33–4.05)	0.003	1.98 (1.10–3.56)	0.023
No Medical Management	2.42 (1.37–4.28)	0.002	2.27 (1.28–4.04)	0.005
Monthly Income < ¥10,000 (< 1389 USD)	1.50 (0.83–2.72)	0.18	-	-
Age ≥35 Years	1.45 (0.85–2.48)	0.175	-	-
Education (Below College)	1.71 (0.99–2.94)	0.053	1.48 (0.86–2.55)	0.157
Assisted Reproduction	1.65 (0.87–3.13)	0.126	-	-

Note: OR, odds ratio; aOR, adjusted odds ratio; CI, confidence interval. The final model included variables with *p* < 0.1 or clinical relevance; “-” indicates exclusion from multivariate analysis; Exchange rate: 1 USD ≈ 7.20 CNY (based on the average exchange rate during the study period).

## Discussion

This study systematically evaluated the combined impact of fetal loss, a major perinatal adverse event, on maternal mental health through a single-center retrospective cohort analysis using PSM. The findings highlight a substantial psychological burden: Mothers experiencing fetal loss had 2.84-fold higher odds of depression compared to those with term delivery, and 72.4% of depression-positive mothers with depression did not receive any form of professional clinical management. These findings not only quantify the psychological impact of perinatal bereavement but also provide key evidence for the construction of an accurate clinical management system by identifying the characteristic clinical phase of “self-blame and fear of re-pregnancy”, as well as key risk factors such as low social support, a history of recurrent miscarriage, and the “lack of clinical management” itself.

The present study found that after PSM to balance baseline confounders, the incidence of depression at 42 days postpartum was still several times higher in mothers with fetal loss than in term delivery, and the risk ratios had clear independence. This finding is consistent with the worldwide research consensus that fetal loss is consistently identified as one of the strongest correlates of postpartum depression [28]. Mainali *et al*. [29] showed that previous perinatal loss was positively associated with both anxiety and depression. This study, using its rigorous methodological design, further strengthens the evidence-based case for including mothers with fetal loss as a population with consistently elevated depression risk requiring routine psychological assessment. The average EPDS score of this population (13.1) exceeded the threshold of clinical screening threshold, suggesting that the psychological distress of this population is universal and clinically significant, and is not a transient emotional response that can be ignored. This quantitative evidence suggests that mental health clinical management for this population may benefit from shifting from a passive, complaint-based model to an active, systematic screening and monitoring model. In this study, the high prevalence of “self-blame” (68.9%) and “fear of future-pregnancy” (62.9%) accurately characterized the unique psychological features of PPD. As a kind of fixed cognitive distortion pointing to clear negative events, the prevalence of “self-blame” is behind complex psychosocial mechanisms. The analysis of the World Health Organization points out that public health information often overplaces the responsibility for pregnancy health over the individual behaviour of pregnant women, which leads to the fact that after the occurrence of unexplained fetal loss, the mother is easy to fall into the internal attribution and produce a strong sense of shame and guilt [30]. Our results align with this explanatory mechanism. The study of Slot *et al*. [31] showed that self-blame, guilt and loss of term delivery after fetal loss were the most prominent emotional experiences of such patients. A study by Gardanova *et al*. [32] on women with recurrent miscarriage found that their most reported feelings on the depression scale were “I feel like a failure” and “I have lost control of my life”. Meanwhile, the study by Balle *et al*. [33] directly quantified “self-blame” as a core predictor of depression. This further confirms its central role in the pathophysiology. Therefore, clinical management targeting this symptom must go beyond general supportive talk to deeply engage in cognitive reconstruction to correct this false self-attribution solidified by the traumatic event. “Fear of re-pregnancy” reveals the extended impact of trauma. The qualitative study by Maryam *et al*. [34] profoundly describes the core of such future catastrophizing expectations with the phrase “If it happened once, it may happen again”. This is not ordinary reproductive anxiety, but an anxiety disorder with avoidant characteristics that is bound to specific traumatic memories. These symptom patterns align closely with established theoretical frameworks of grief and trauma. The prominent self-blame observed in our cohort reflects maladaptive grief-related cognitions characteristic of complicated grief [26], while fear or avoidance of a future pregnancy can be conceptualized as a trauma-specific cognitive bias consistent with post-traumatic stress disorder [29]. Situating our findings within these frameworks not only deepens the theoretical understanding of post-loss psychological distress but also points to specific, empirically supported intervention targets. This requires that clinical management must include post-loss fertility counselling as a standard component aimed at dealing with traumatic memories, reducing anxiety sensitivity, and helping mothers rebuild a sense of psychological safety and control for future reproductive choices.

Within the population with fetal loss, we developed a multilevel risk model, whose findings both echoed and deepened existing evidence. This study confirmed that the history of recurrent miscarriage was an independent risk factor, which was consistent with the conclusion of large cohort studies that regarded “multiple fetal loss” as a marker of long-term psychological trauma [35]. Low social support was the strongest risk factor in this study, which was fully consistent with the stress buffer theory. The study of Hu *et al*. [36] provided a mechanistic explanation for this, and found that perceived social support can significantly buffer the negative effects of abortion events on depression and anxiety symptoms. Several studies have confirmed that support from spouses, family and friends can significantly reduce the risk of postpartum depression [37, 38]. This emphasizes that clinical intervention must leap from the individual level to the relationship and system level and actively mobilize and guide partners and families to provide effective support. In addition, not receiving clinical management was independently associated with depression. Previous studies mostly focused on the effectiveness of approaches, while this study quantified the direct harm of the “treatment gap” itself on individual outcomes. Our finding of a high rate of non-receipt of clinical management (72.4%) starkly contrasts with a study by Nynas *et al*. [39], which reported that while 90% of women after miscarriage desired specific follow-up care, only 30% actually received it. The study of Kong *et al*. [18] also revealed that medical professionals had a lower understanding of the psychological impact of abortion than postpartum depression, and believed that medical professionals should pay more attention to the psychological diseases related to abortion and should provide routine psychological intervention. This highlights systemic gaps in identification and referral as key modifiable factors associated with prolonged patient distress.

The most urgent call to action stems from this study’s revelation: the effectiveness of clinical management was substantial (those who received clinical management showed nearly three times greater improvement than those who did not), but its current rate of use is unacceptably low. This efficacy finding is not unique. The randomized controlled trial of Nasrollahi *et al*. [16] confirmed that the implementation of structured “mindfulness-based stress reduction” intervention for women with fetal loss can significantly improve their psychological state. It is more fitting that Johnson *et al*. [17] published the international frontier standardized interpersonal psychotherapy (IPT) for perinatal bereavement, whose preset goals are “reducing the fear of re-pregnancy” and “enhancing social support”. This is highly consistent with the need for intervention revealed in this study. Collectively, this evidence suggests that effective approaches exist and that a systematic approach to integrating them into routine care is the central paradox. However, these recommendations are derived from associative findings rather than causal evidence, given the retrospective, single-centre, and non-randomized design of the present study. The observed associations—while robust and clinically meaningful—do not establish causality. Therefore, the proposal of institutionalized, systematic mental health pathways should be interpreted as a hypothesis-generating direction supported by the current data, pending confirmation in prospective cohort studies and randomized controlled trials.

As a retrospective study, this study has inherent limitations. First, while PSM balanced known confounders, matching was limited to demographic and obstetric variables available in the database. Psychosocial factors (e.g., social support, marital satisfaction) were measured only post-loss and thus could not be included in matching, and residual confounding may persist. Second, these psychosocial factors were assessed concurrently with depressive symptoms at 42 days post-loss; therefore, reverse causality (e.g., depressed mood negatively influencing perceived support) cannot be ruled out. Third, this single-centre study was conducted within a specific sociocultural and healthcare context, limiting generalizability to other settings. Fourth, the 3-month follow-up captures early psychological responses but does not reflect long-term trajectories of depression. Fifth, fetal loss includes heterogeneous conditions (e.g., missed abortion, inevitable abortion, stillbirth) that may differ in psychological impact; due to limited subgroup sample sizes, stratified analyses by loss type or gestational age were not feasible. Sixth, receipt of clinical management was non-randomized and may have been influenced by baseline severity or help-seeking behaviour; thus, the observed association between receipt of clinical management and symptom improvement should not be interpreted as causal. Although we adjusted for baseline EPDS and observed no significant differences in measured covariates, residual confounding and regression to the mean cannot be entirely excluded. Seventh, depression was defined by a single EPDS assessment at 42 days using the conventional ≥13 cut-off. This threshold has limited validation specifically in post-loss populations, and some participants classified as depressed may have been experiencing transient grief rather than persistent depressive disorder. Moreover, depression caseness was not reassessed at 3 months, limiting interpretation of intervention effectiveness: while those who received clinical management showed greater symptom reduction, whether receipt of clinical management reduced the incidence of clinical depression remains unknown. Based on our findings, we propose the following directions for future research: First, enrolling a broader and more diverse population, with baseline assessments starting in the first trimester and prospectively following up to 1 to 2 years or more after fetal loss. It can describe the trajectory of psychological risk more accurately and establish a depression risk prediction model including biological, psychological and social factors. Second, a rigorous RCT was designed and implemented to verify the efficacy of specific psychological clinical management targeting self-blame and fear of repregnancy. Thirdly, the cost-effectiveness of a systematic psychological screening and intervention program compared with usual care was evaluated. Providing clear economic evidence is key to integrating such services into public health policy and winning support from policymakers.

## Conclusions

This study identifies fetal loss as a significant independent correlate of postnatal depression, presenting distinct clinical manifestations. The associated risk is strongly influenced by social support, marital quality, obstetric history, and healthcare engagement. The findings further suggest that “lack of timely and standardized professional care” may be a key factor associated with the psychological distress and worsening long-term prognosis of mothers with fetal loss, which reflects a potential gap in current clinical practice. These results support the consideration of institutionalized, systematic, evidence-based mental health pathways for this vulnerable population. However, given the observational design, causal inference requires confirmation in prospective and interventional studies.

## Availability of Data and Materials

The data supporting the findings of this study can be obtained from the corresponding author, upon request.
